# Parrotfish grazing ability: interspecific differences in relation to jaw-lever mechanics and relative weight of adductor mandibulae on an Okinawan coral reef

**DOI:** 10.7717/peerj.2425

**Published:** 2016-09-01

**Authors:** Atsushi Nanami

**Affiliations:** Research Center of Sub-tropical Fisheries, Seikai National Fisheries Research Institute, Japan Fisheries Research and Education Agency, Ishigaki, Okinawa, Japan

**Keywords:** Parrotfish, *Chlorurus*, *Scarus*, Grazing ability, Interspecific difference, Excavator, Scraper, Jaw-lever mechanics

## Abstract

Parrotfishes (family Labridae: Scarini) are regarded to have important roles for maintaining the ecosystem balance in coral reefs due to their removal of organic matter and calcic substrates by grazing. The purpose of the present study was to clarify the interspecific differences in grazing ability of five parrotfish species (*Chlorurus sordidus*, *C. bowersi*, *Scarus rivulatus*, *S. niger* and *S. forsteni*) in relation to interspecific differences in jaw-lever mechanics and the relative weight of the adductor mandibulae (muscles operating jaw closing). The grazing ability was calculated by using stomach contents (CaCO_3_ weight/organic matter weight) defined as the grazing ability index (GAI). There were significant interspecific differences in GAI (*C. sordidus* = *C. bowersi* > *S. rivulatus* > *S. niger* = *S. forsteni*). Teeth of *C. sordidus* and *C. bowersi* were protrusive-shape whereas teeth of *S. rivulatus*, *S. niger* and *S. forsteni* were flat-shape. *C. sordidus* and* C. bowersi*have jaw-lever mechanics producing a greater biting force and have a larger weight of adductor mandibulae. *S. rivulatus* has jaw-lever mechanics producing a greater biting force but a smaller weight of adductor mandibulae that produce an intermediate biting force. In contrast, *S. niger* and *S. forsteni* have jaw-lever mechanics producing a lesser biting force and have a smaller weight of adductor mandibulae. Feeding rates and foray size of *S. rivulatu*s, *S. niger* and *S. forsteni* were greater than * C. sordidus* and* C. bowersi*. The degree in bioerosion (GAI × feeding rate) was the largest for *S. rivulatus*and the smallest for *S. forsteni*. The degree in bioerosion for *C. sordidus* was larger than *S. niger* whereas relatively equal between *C. bowersi* and *S. niger*. These results suggest that interspecific difference in GAI was explained by interspecific differences in teeth shape, jaw-lever mechanics and relative weight of adductor mandibulae. The interspecific difference in the degree of bioerosion suggests the importance of various size of parrotfishes with diverse feeding modes to maintain healthy coral reef ecosystems.

## Introduction

Coral reefs support high species diversity of marine organisms including fishes. Among the diverse species of coral reef fishes, parrotfishes (family Labridae: Scarini) are considered to be important components to maintain a healthy coral reef ecosystem in relation to their feeding mode (reviewed in Bonaldo, Hoey & Bellwood, 2014). Parrotfishes are considered to contribute to the enhancement of settlement of benthic organisms including corals by removing epilithic algae and benthic organisms on the substrates by gazing (Mumby et al., 2006; Adams et al., 2011 but also see Russ et al., 2015). Parrotfishes are also considered to contribute to the bioerosion of coral reefs which is an important process to build coral reef environments (Bellwood, 1995a; Bellwood, 1995b). A decrease in parrotfish density due to human activities would subsequently cause dramatic change in the ecosystem balance and resilience of coral reefs (Bellwood et al., 2004; Bellwood, Hoey & Hughes, 2011; Hughes et al., 2007).

Bellwood & Choat (1990) categorized parrotfishes into two functional groups based on the morphological characteristics of jaws (premaxilla, maxilla, dentary and articular). The first group is formed of excavators that have a deep shape and a thick cement covering of jaws. Feeding behavior of excavators shows a short powerful bite and the degree of grazing was greater, which was found by their bite marks termed as scars (Bellwood, 1995a; Bellwood, 1995b; Bellwood, 1996; Alwany, Thaler & Stachowitsch, 2009; Bonaldo & Bellwood, 2009). Excavators mainly consist of genus *Bolbometopon*, *Cetoscarus*, *Chlorurus* and some *Sparisoma* species (Bellwood & Choat, 1990; Bellwood, 1994). The second group is formed of scrapers that have a shallow shape and thin cement covering of jaws. Scrapers deliver weaker bites and graze less, per bite unit, than excavators (Lokrantz et al., 2008; Bonaldo, Krajewski & Bellwood, 2011). Scrapers mainly consist of the genus *Scarus* and several *Sparisoma* species (Bellwood & Choat, 1990; Bellwood, 1994).

Some previous studies have estimated the grazing ability of parrotfishes by observation of scars (Bellwood, 1995a; Bellwood, 1995b; Bellwood, 1996; Van Rooij, Videler & Bruggemann, 1998; Alwany, Thaler & Stachowitsch, 2009; Bonaldo & Bellwood, 2009; Bonaldo, Krajewski & Bellwood, 2011). Scars are found when parrotfishes graze the substrates for feeding and the characteristics of the scars were different between excavators and scrapers in relation to their jaw morphology (e.g., Alwany, Thaler & Stachowitsch, 2009; Bonaldo & Bellwood, 2008; Bonaldo & Bellwood, 2009; Lokrantz et al., 2008; Bonaldo, Krajewski & Bellwood, 2011). These studies measured the size of scars (depth × width × length) for quantitative estimations of the grazing ability and showed that the size of scars was greater for excavators than scrapers (Hoey & Bellwood, 2008; Lokrantz et al., 2008; Bonaldo & Bellwood, 2009). Several studies have also shown that the larger size of scars was found for larger size of individuals, indicating the ontogenetic difference in grazing ability of parrotfishes (Bruggemann et al., 1996; Bonaldo & Bellwood, 2008; Lokrantz et al., 2008).

Bruggemann et al. (1996) also estimated the grazing ability of two parrotfish species based on the stomach contents and showed that the grazing ability of excavator species was greater than scraper species. Ontogenetic differences in grazing ability were also found for the two species: the smaller-sized individuals (less than 14 cm in fork length) did not graze on the carbonate substrates and removed only epilithic algae, whereas larger-sized individuals (over 15 cm in fork length) ingested the calcium carbonate substrates during grazing events. Thus, Bruggemann et al. (1996) also showed that the feeding mode and body size are good indicators of the degree of grazing ability of parrotfishes.

Although parrotfishes are major fish assemblage components in Okinawan coral reefs, ecological studies on grazing and bioerosion focusing on interspecific differences in jaw-lever mechanics and relative weight of adductor mandibulae (muscle operating jaw closing) have not been clarified yet in this region (Bonaldo, Hoey & Bellwood, 2014). In addition, although jaw-lever mechanics and relative weight of adductor mandibulae are the key aspects to clarify the feeding ecology of marine fishes, quantitative comparisons of interspecific differences in jaw-lever mechanics and relative weight of adductor mandibulae have not been sufficiently examined for multiple species of parrotfishes.

The aims of the present study were to investigate the grazing ability of five species of parrotfishes in an Okinawan coral reef. Specifically, the aims were to clarify (1) interspecific differences in grazing ability, (2) ontogenetic variations in grazing ability, (3) a quantitative description of jaw-lever mechanics, (4) an interspecific comparison in relative adductor mandibulae weight to parrotfish size and (5) the feeding behavior of parrotfishes in this region.

## Materials and Methods

The study was conducted mainly using field observations of free-living fishes in their natural habitat. Individuals caught for sampling were immediately killed by placing them on ice to minimize pain. The sampling procedure was approved by Okinawa prefectural government fisheries coordination regulation No. 41, which permits capture of marine fishes on Okinawan coral reefs for scientific purposes.

### Study species

**Figure 1:**
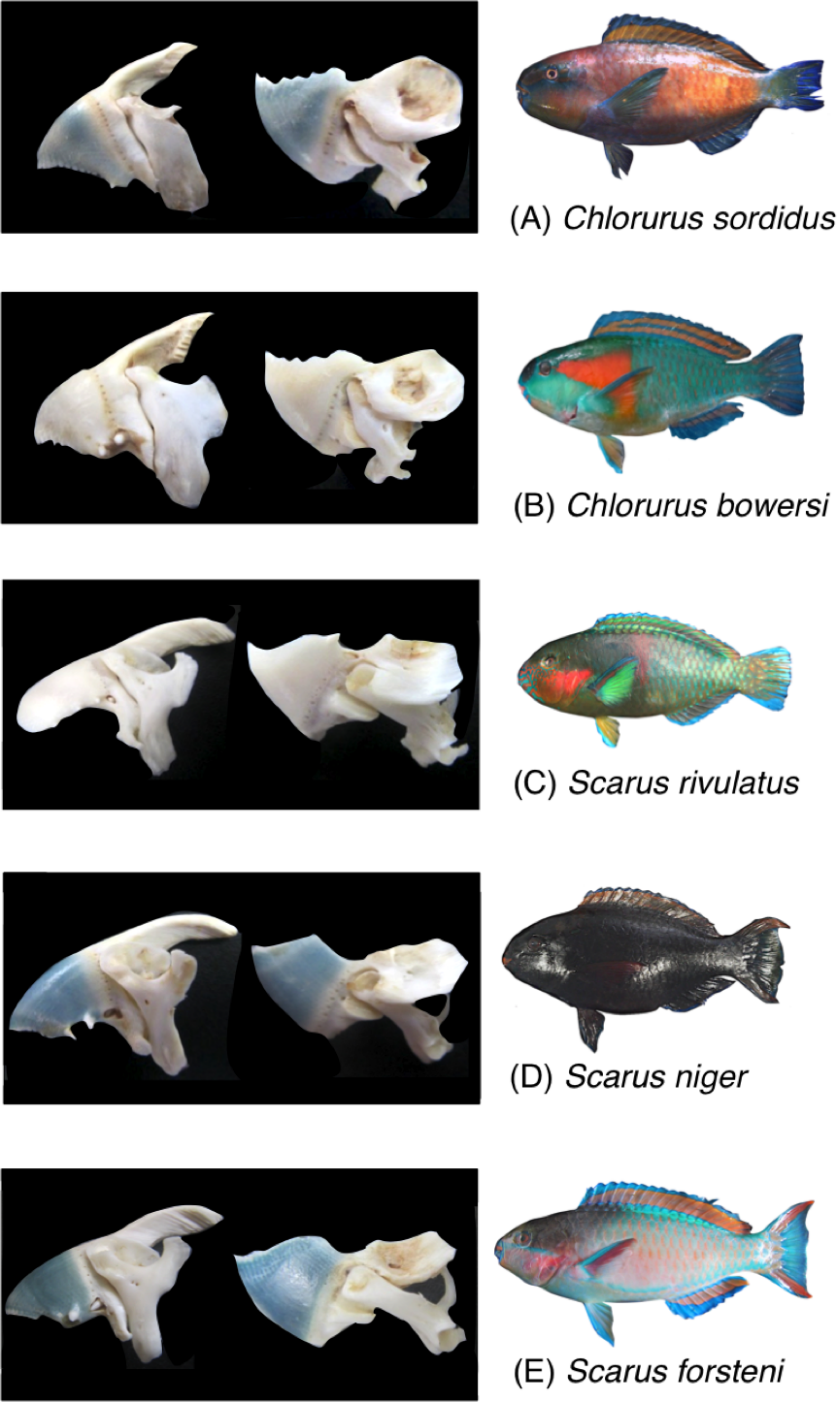
Photographs of the five parrotfish species with their upper jaw lever-system (premaxilla + maxilla) and lower jaw lever system (dentary + articular).

Five parrotfish species were selected for the present study (*Chlorurus sordidus*, *C. bowersi*, *Scarus rivulatus*, *S. niger* and *S. forsteni*) (Fig. 1). These five species are commonly found in Okinawa.

### Estimation of grazing ability using stomach contents

**Figure 2:**
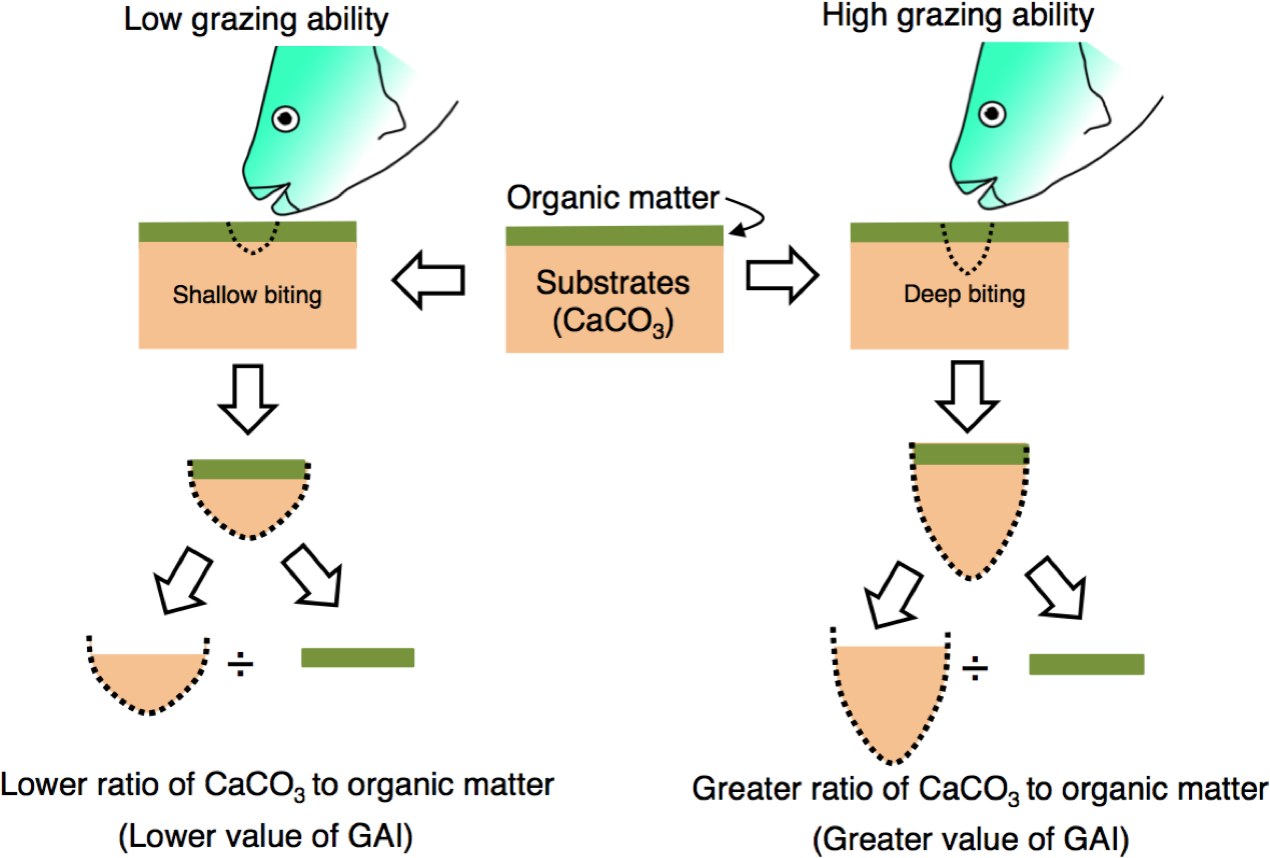
Schematic diagram of the definition of grazing ability index (GAI). Substrates consist of calcium carbonate (CaCO_3)_ and are covered by organic matter (epilithic algae, sensu Bellwood & Choat, 1990). The ratio of CaCO_3_ weight to organic matter weight would change with the difference in grazing ability. For measurement procedures of the CaCO_3_ weight and organic matter weight, see ‘Materials and Methods’.

The grazing ability of parrotfish was estimated from stomach contents as follows. Since the substrate of coral reefs consists of limestone, the stomach contents of parrotfish can be considered to contain the calcium carbonate (CaCO_3_). Namely, the stomach contents can be regarded as mixtures of organic matter (e.g., epilithic algae) and CaCO_3_ (stomach contents = organic matter + CaCO_3_) (Fig. 2). In the present study, it was considered that the ratio of CaCO_3_ in the stomach contents would be higher if the grazing ability is greater (i.e., deep biting, Fig. 2). Therefore, the ratio (CaCO_3_ weight/organic matter weight) is defined as the “grazing ability index” (GAI) in the present study.

The CaCO_3_ weight was calculated as:

\begin{eqnarray*}{\mathrm{CaCO}}_{3}\mathrm{weight}=(\text{stomach content weight})-(\text{organic matter weight}). \end{eqnarray*}

CaCO_3_ can be removed from the stomach contents by adding hydrochloric acid (HCl) aqueous solution as the following reaction formula:

\begin{eqnarray*}{\mathrm{CaCO}}_{3}+2\mathrm{HCl}\Rightarrow {\mathrm{CaCl}}_{2}+{\mathrm{H}}_{2}\mathrm{O}+{\mathrm{CO}}_{2} \end{eqnarray*}

where CaCl_2_ is calcium chloride, H_2_O is water and CO_2_ is carbon dioxide. Since CaCl_2_ is water-soluble and CO_2_ isvolatile, the CaCO_3_ as well as CaCl_2_ and CO_2_ were completely removed from the stomach contents by water rinsing. Then, the ratio of CaCO_3_ to organic matter weight (i.e., GAI) was calculated as:

\begin{eqnarray*}\mathrm{GAI}=({\text{dry weight of CaCO}}_{3})/(\text{dry weight of organic matter})=[\text{dry weight of}(\text{stomach content}-\text{organic matter})]/[(\text{dry weight of organic matter})]. \end{eqnarray*}

Thus, the GAI represents the CaCO_3_ weight that was simultaneously grazed per unit of weight of organic matter on substrate.

### Sampling and experimental procedure

For the GAI estimation, five parrotfish species were sampled by spear gun between July 2013 and February 2016 from the reef slope at Ishigaki Island, Okinawa, Japan. The number of individuals and the range of fork length (FL) were as follows: *C. sordidus* (*n* = 33: 149.5–236.0 mm), *C. bowersi* (*n* = 23: 179.5–265.5 mm), *S. rivulatus* (*n* = 26: 154.0 mm–319.5 mm), *S. niger* (*n* = 20: 166.0–288.0 mm) and *S. forsteni* (*n* = 22: 145.0–303.5 mm). All fishes were collected by spearing the head and immediately killed in an icebox to minimize any loss of stomach contents.

Dry weight in the stomach contents and organic matter were determined as follows: (1) a subsample of stomach contents (c.a. 3 g in wet weight) was put into a 1.5 ml microtube and dried at 58 °C; (2) the weight of the dried stomach contents was measured to the nearest 0.0001 g; (3) 1N-HCl aqueous solution was poured onto the stomach contents; (4) the microtube was centrifuged and supernatant HCl aqueous solution was removed using micro-pipet with care not to remove any stomach contents; (5) the third and fourth procedures were repeated until no CO_2_ bubbles were found during the third procedure; (6) the remaining stomach contents were rinsed using distilled water, centrifuged and the supernatant water was removed. Finally, the organic matter (stomach contents with no CaCO_3_) was dried at 58 °C and the weight was measured to the nearest 0.0001 g.

### Interspecific comparison in GAI

The relationships between FL and GAI were plotted as a logarithmic function as:

\begin{eqnarray*}\mathrm{GAI}=a\text{ }{\log \nolimits }_{\mathrm{e}}(\mathrm{FL})+b \end{eqnarray*}

where *a* and *b* are coefficients. One-way ANOVA was conducted for interspecific comparisons of GAI for four FL ranges with 40-mm intervals: (1) 140.0 mm–180.0 mm, (2) 180.5 mm–220.0 mm, (3) 220.5 mm–260.0 mm and (4) over 260.5 mm. If a significant difference was found, a post-hoc Games-Howell test was applied for multiple comparisons among the five species.

### Teeth characteristics

Number of teeth on dental plates for premaxilla and dentary was counted. A total of 10 samples were used for each species. One-way ANOVA and post-hoc Games-Howell test was used for significant differences in the ratio among the five species.

### Jaw-lever mechanics for closing

**Figure 3:**
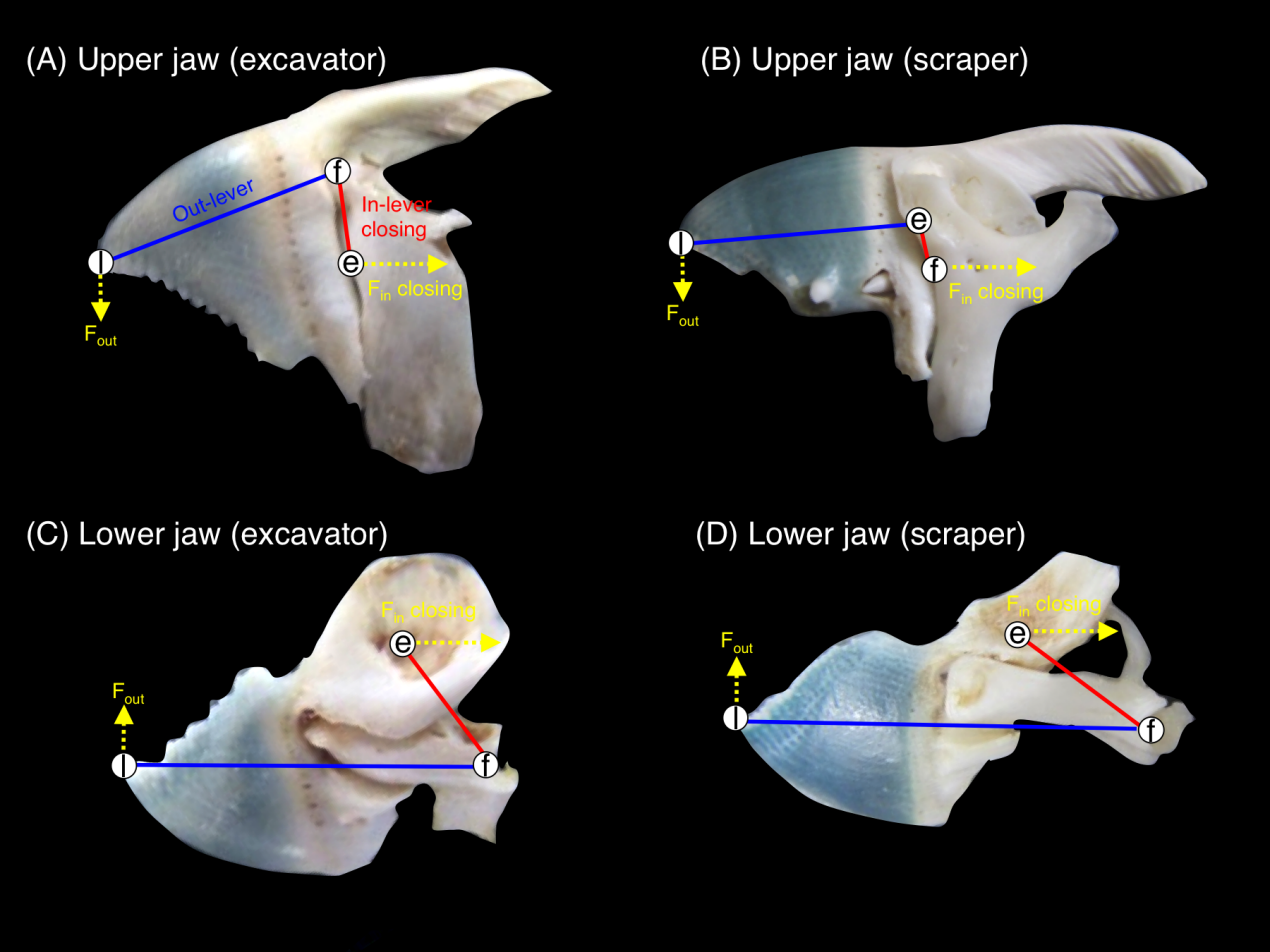
Pictorial explanation of the jaw-lever mechanics for closing (e, effort; f, fulcrum; l, load. See also Fig. S1). (A) upper jaw of excavator (*Chlorurus sordidus*); (B) upper jaw of scraper (*Scarus forsteni*); (C) lower jaw of excavator (*Chlorurus sordidus*); (D) lower jaw of scraper (*Scarus forsteni*). The relative difference in biting force and velocity can be estimated by measuring the length of the in-lever-closing (red lines: L_in-lever-closing_) and out-lever (blue lines: L_out-lever_). The ratio of in-lever-closing to out-lever (L_in-lever-closing_/ L_out-lever_) can be calculated in accordance with Wainwright & Bellwood (2002), Westneat (2004) and Westneat et al. (2005). The greater values in the ratio means greater biting force but lower velocity in biting and vice versa. F_in-closing_: force for jaw closing at tendon (see also Fig. S1). F_out_: produced force for jaw rotation.

Jaw-lever mechanics for closing was compared by measuring the morphological characteristics of the upper jaw (premaxilla + maxilla) and lower jaw (dentary + articular) in accordance with Wainwright & Bellwood (2002), Westneat (2004) and Westneat et al. (2005). Jaw-lever mechanics are related to the force and velocity in the feeding of fishes (see Westneat, 2004; Westneat et al., 2005). In accordance with Bellwood & Choat (1990), the jaw-lever mechanics for closing was investigated (Fig. S1). The length of in-lever-closing (L_in-lever-closing_) and out-lever (L_out-lever_) were measured by using a digital caliper (Fig. 3). Then, the ratio of in-lever-closing to out-lever (L_in-lever-closing_/L_out-lever_) were calculated and compared among the five species. The greater values in the ratio means greater biting force but lower velocity in biting and vice versa (Westneat, 2004). A total of 10 samples were used for each species. One-way ANOVA and post-hoc Games-Howell test was used to test for significant differences in the ratio among the five species.

### Muscles operating the jaw

Weight of muscles operating jaw (adductor mandibulae) was measured nearest 0.01 g by dissecting. The relationship between FL and adductor mandibulae weight was plotted as a power function as:

\begin{eqnarray*}\text{adductor mandibulae weight}=a\text{ }{\mathrm{FL}}^{b} \end{eqnarray*}

where *a* and *b* are coefficients. Analysis of covariance (ANCOVA) was performed to compare the inter-specific difference in the FL- adductor mandibulae weight relationship. Prior to the analysis, the data was log-transformed and the relationship was converted as a simple linear regression. If a significant difference was found, a post-hoc Bonferroni test was applied for multiple comparisons among the five species.

### Underwater observations of feeding behavior

Feeding behavior was observed at the fringing reef around Ishigaki Island between October and November 2011 and between October 2012 and February 2013. Underwater observations were conducted at the reef slope (fore reef) using SCUBA equipment or snorkeling. Depth range was 3 m–5 m and water temperature was 22 °C–28 °C. A researcher (A Nanami) searched for individuals of the focal species and followed a focal individual paying particular attention not to disturb the behavior of the individual. To avoid double-counts for the focal individual, species, size and/or sex were changed for the subsequent observations. The estimated FL (cm), number of bites per 5 min (feeding rate) and foray size (mean number of bites: sensu Bellwood & Choat, 1990) were counted. The FL range was between 25 cm and 35 cm and time zone was between 1200 h–1600 h. Since previous studies have demonstrated a time-zone difference in feeding rates (e.g., 0800 h–1000 h, 1001 h–1200 h, 1201 h–1400 h and 1401 h–1600 h; Bellwood, 1995a; Bonaldo & Bellwood, 2008), a one-way ANOVA was performed to clarify if any time-zone differences occurred in the feeding rates for the two time zones (1200 h–1400 h and 1401 h–1600 h). As a result, no significant time-zone differences were found for all five species. Thus, the feeding rates obtained in the two time zones were pooled and used for further analyses. The numbers of individuals were 10, 11, 11, 7 and 8 for *Chlorurus sordidus*, *C. bowersi*, *Scarus rivulatus*, *S. niger* and *S. forsteni*, respectively. One-way ANOVA and a post-hoc Games-Howell test were applied for multiple comparison for feeding rates and foray size among the five species.

During the observations of feeding behavior, the numbers of bites per substrate were also recorded. Previous studies have categorized the feeding substrates as epilithic algae on hard substrata, macroalgae, live coral, crustose coralline algae and seagrass (Rotjan & Lewis, 2005; Rotjan & Lewis, 2009; Hoey & Bellwood, 2008; Alwany, Thaler & Stachowitsch, 2009; Nash et al., 2012; Bonaldo, Hoey & Bellwood, 2014). The data for the respecting feeding substrates were represented as the proportion of the number of bites (%) for each substrate against the total number of bites. The proportions of bites for each individual were averaged for each species.

### Interspecific comparison in degree of bioerosion

From the data of GAI and feeding rates, the degree of bioerosion was calculated as follows:

\begin{eqnarray*}\text{degree of bioerosion}=(\mathrm{GAI})\times (\text{feeding rate}). \end{eqnarray*}

This was because the degree of bioerosion should be considered by both GAI and feeding rates. For calculation of the degree of bioerosion, calculated GAI data at 30 cm FL were obtained using the above-mentioned FL-GAI relationship. The rate of focal species to another species was calculated for all pairs of the five species for interspecific comparison of the degree of bioerosion.

## Results

### Interspecific difference in GAI

**Figure 4:**
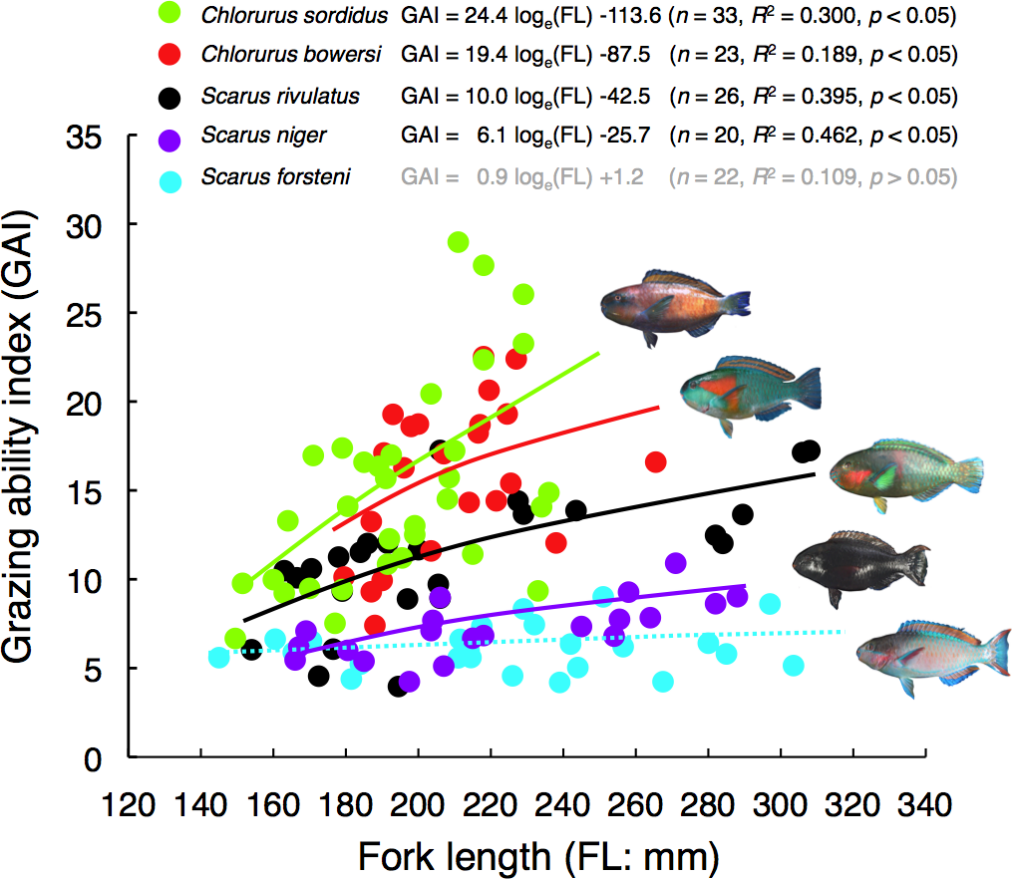
Relationships between fork length (FL) and grazing ability index (GAI) for the five parrotfish species. The relationships are plotted by logarithmic function.

**Figure 5:**
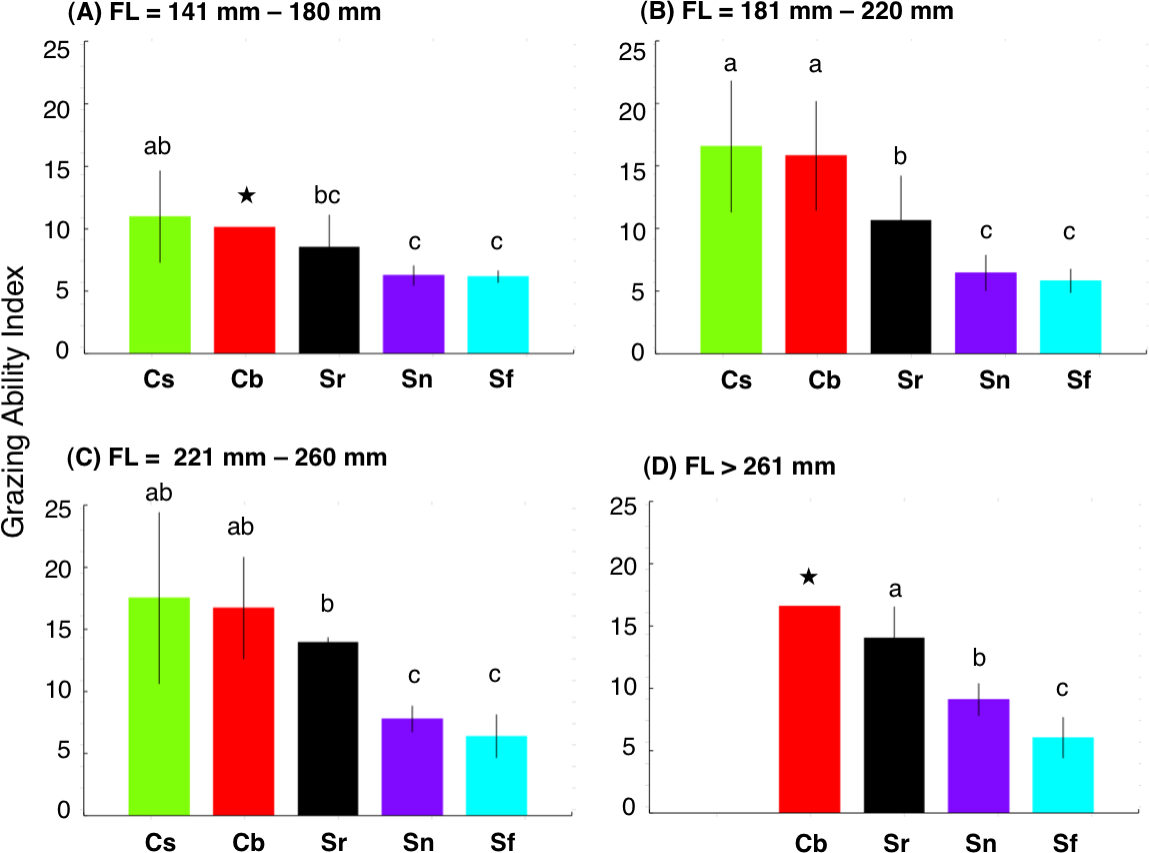
Interspecific difference in grazing ability index (GAI) for four fork length (FL) ranges. The different alphabet characters on the bars indicate the significant difference in GAI (Games-Howell test, *p* < 0.05). Vertical lines represent standard deviation. Star symbols in (A) and (D): only 1 individual for *Chlorurus bowersi* and excluded from Games-Howell test due to no standard deviations. No individuals for *C. sordidus* in (d). Species names are abbreviated as Cs, *Chlorurus sordidus*; Cb, *C. bowersi*; Sr, *Scarus rivulatus*; Sn, *S. niger*; Sf, *S. forsteni*.

There were significant positive relationships between FL and GAI for four species (*Chlorurus sordidus*, *C. bowersi*, *Scarus rivulatus* and *S. niger*) (Fig. 4). Although a slight positive relationship between FL and GAI was found for *S. forsteni*, it was not significant (Fig. 4). Overall, the GAIs were greater for *C. sordidus* and *C. bowersi*, intermediate for *S. rivulatus* and lesser for *S. niger* and *S. forsteni* (Fig. 5). For the 141 mm–180 mm FL range, the GAI of *C. sordidus* was significantly greater than *S. niger* and *S. forsteni* (Games-Howell test, *p* < 0.05) (Fig. 5A). For the 181–220 mm FL range, GAIs of *C. sordidus* and *C. bowersi* were significantly greater than the other three species (Fig. 5B). The GAI of *S. rivulatus* was also significantly greater than *S. niger* and *S. forsteni* (Fig. 5B). For the 221 mm–260 mm FL range, the GAIs for *C. sordidus*, *C. bowersi* and S. *rivulatus* were significantly greater than *S. niger* and *S. forsteni* (Fig. 5C). For the over 260 mm FL range, significant differences in GAI were found among *S. rivulatus*, *S. niger* and *S. forsteni* (Fig. 5D).

### Interspecific differences in teeth characteristics

**Figure 6:**
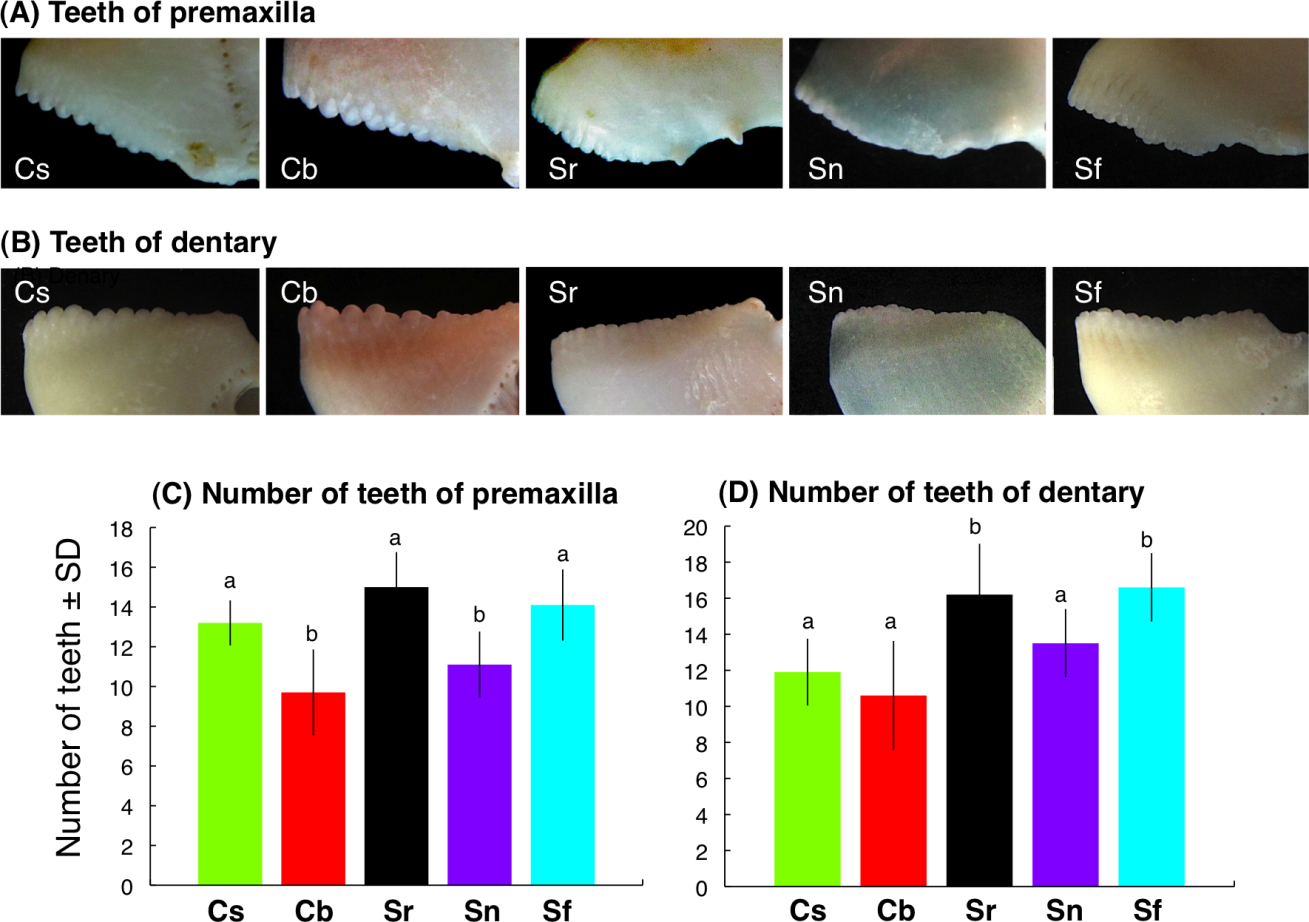
Photographs of teeth on dental plates of premaxilla (A) and dentary (B). Number of teeth on dental plates of premaxilla (C) and dentary (D). Different alphabet characters on the bars indicate significant difference (one-way ANOVA and post-hoc Games-Howell test, *p* < 0.05). Species names are abbreviated as Cs, *Chlorurus sordidus*; Cb, *C. bowers*i; Sr, *Scarus rivulatus*; Sn, S. *niger*; Sf, *S. forsteni*.

Each tooth was protrusive-shape for *C. sordidus* and *C. bowersi* whereas each tooth was relatively flat-shape for *S. rivulatus*, *S. niger* and *S. forsteni* for premaxilla (Fig. 6A) and dentary (Fig. 6B). The number of teeth of premaxilla for *C. sordidus*, *S. rivulatus* and *S. forsteni* was significantly greater than *C. bowersi* and *S. niger* (Fig. 6C). The number of teeth of dentary for *S. rivulatus* and *S. forsteni* was significantly greater than *C. sordidus*, *C. bowersi* and *S. niger* (Fig. 6D).

### Interspecific differences in jaw-lever mechanics for closing

**Figure 7:**
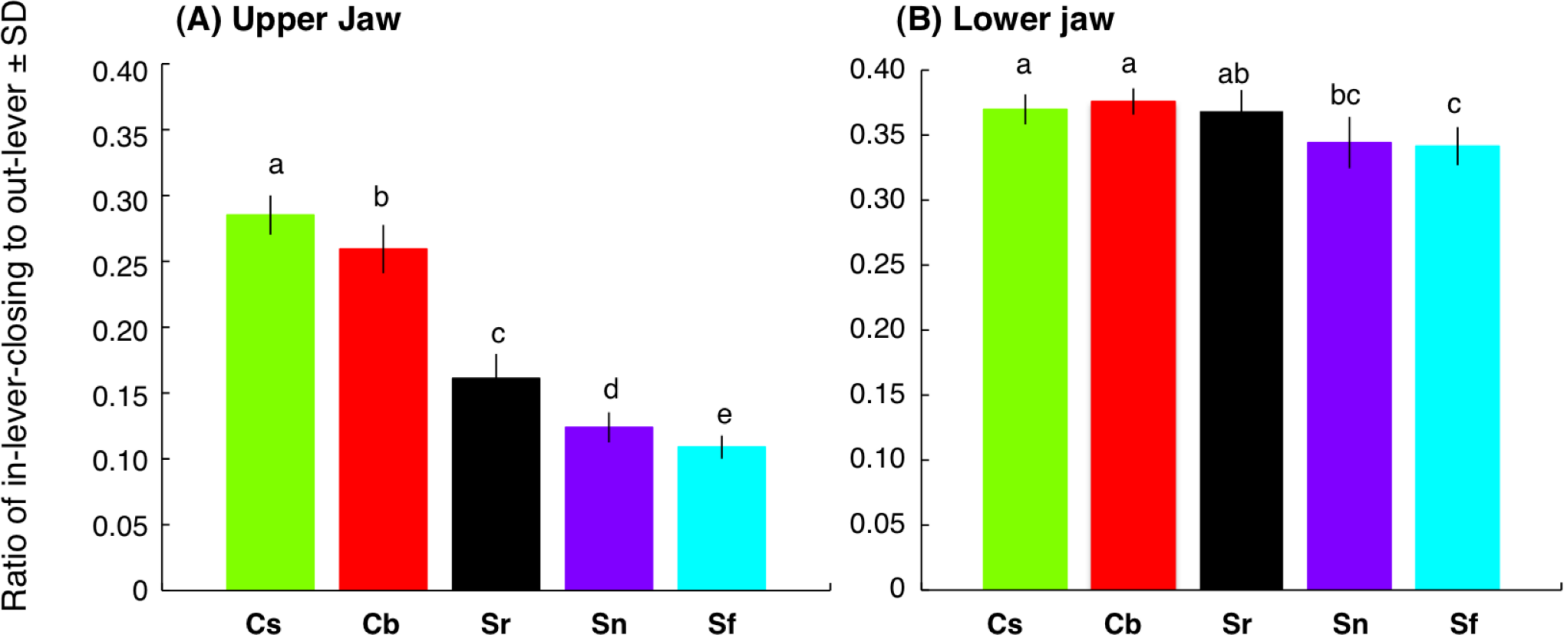
Interspecific difference in the ratio of in-lever-closing to out-lever (L_in-lever-closing_/ L_out-lever_) (see also Fig. 3) for upper jaw (A) and lower jaw (B). The greater values in the ratio means higher force but lower velocity, whereas lower values in the ratio means higher velocity but lower force (Westneat, 2004). Different alphabet characters on the bars indicate significant difference (one-way ANOVA and post-hoc Games- Howell test, *p* < 0.05). Species names are abbreviated as Cs, *Chlorurus sordidus*; Cb, *C. bowersi*; Sr, *Scarus rivulatus*; Sn, *S. niger*; Sf, *S. forsteni*.

For the upper jaw, the ratio of in-lever-closing to out-lever (L_in-lever-closing_/L_out_) was significantly different among the five species (*C. sordidus* > *C. bowersi* > *S. rivulatus* > *S. niger* > *S. forsteni*) (Fig. 7A) (one-way ANOVA and post-hoc Games-Howell test, *p* < 0.05). For the lower jaw, the ratio of in-lever-closing to out-lever (L_in-lever-closing_/L_out_) for *C. sordidus* and *C. bowersi* was significantly greater than *S. niger* and *S. forsteni* (Fig. 7B). No significant difference in the ratio was found among *C. sordidus*, *C. bowersi* and *S. rivulatus* and between *S. rivulatus* and *S. niger* (Fig. 7B).

### Interspecific difference in relationship between FL and adductor mandibulae weight

**Figure 8:**
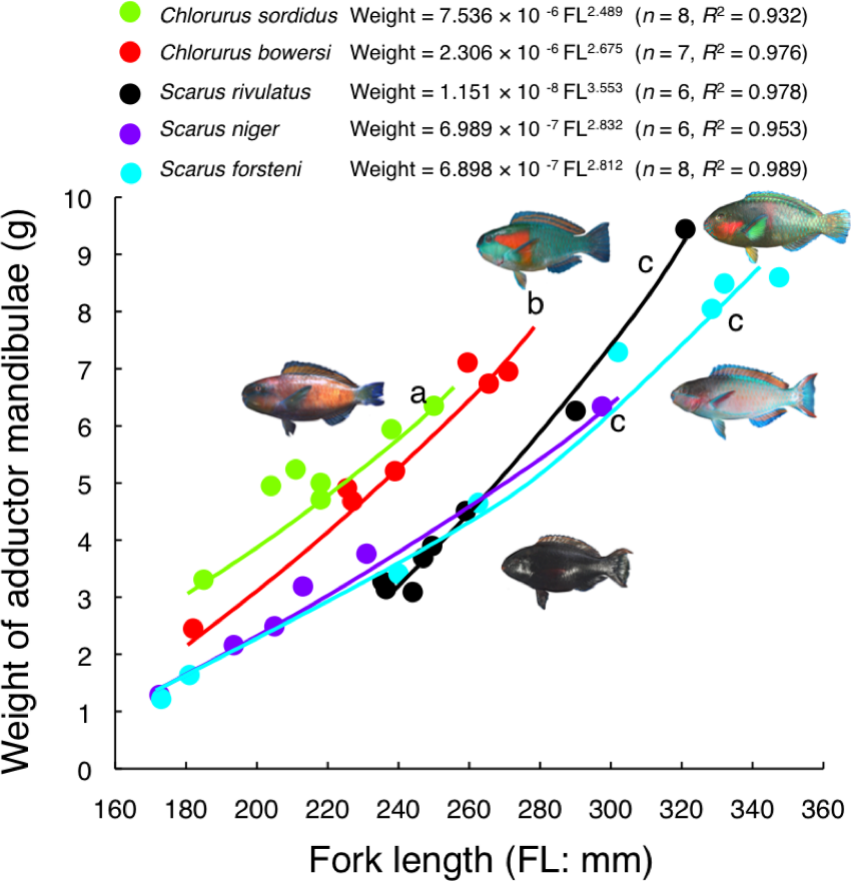
Relationship between fork length and weight of adductor mandibulae for the five parrotfish species. Different alphabet characters near the regression lines indicate significant difference (ANCOVA and post-hoc Bonferroni test, *p* < 0.05).

Relative weight of adductor mandibulae against FL for *C. sordidus* and *C. bowersi* was significantly greater than the other three species (ANCOVA and post-hoc Bonferroni test, *p* < 0.05) (Fig. 8). The relative weight for *C. sordidus* was significantly greater than *C. bowersi*. In contrast, no significant difference was found among *S. rivulatus*, *S. niger* and *S. forsteni* (ANCOVA and post-hoc Bonferroni test, *p* > 0.05) (Fig. 8).

### Feeding behavior

Feeding rates for *Scarus rivulatus*, *S. niger* and *S. forsteni* tended to be higher than *C. sordidus* and *C. bowersi* (Table 1). The feeding rates of *S. rivulatus* and *S. niger* were significantly higher than *C. sordidus* and *C. bowersi* (one-way ANOVA and post-hoc Games-Howell test, *p* < 0.05). A similar tendency was found for foray size (Table 1). The foray size of *S. rivulatus* and *S. niger* were significantly larger than *C. sordidus* and *C. bowersi* (one-way ANOVA and post-hoc Games-Howell test, *p* < 0.05). The main feeding substrate was epilithic algae on hard substrates such as rocks and dead coral skeletons. No individuals were observed feeding on macroalgae, crustose coralline algae or seagrass in the present study. Live coral were rarely fed on by *C. bowersi* and *S. niger* (Table 1). *C. sordidus*, *S. rivulatus* and *S. forsteni* did not feed on live coral.

**Table 1:** Interspecific difference in feeding rate, foray size and location of feeding. For feeding rate and foray size, different alphabet characters attached on the numbers indicate significant differences among species (one-way ANOVA and post-hoc Games-Howell test, *p* < 0.05).

Species	*n*	Feeding rates (5 min^−1^)	Foray size	Proportion of bites (%)	
		±SD	±SD	Epilithic algae	Live coral
*Chlorurus sordidus*	10	75.0 ± 31.7^ab^	6.3 ± 2.1^ac^	100	0
*C. bowersi*	11	66.3 ± 29.0^ab^	6.1 ± 3.5^ac^	99.8	0.2
*Scarus rivulatus*	11	186.0 ± 50.7^c^	15.8 ± 7.8^b^	100	0
*S. niger*	7	158.0 ± 53.3^cd^	15.9 ± 4.6^b^	98.3	1.7
*S. forsteni*	8	111.8 ± 37.9^bd^	9.2 ± 4.7^bc^	100	0

**Notes.**

SD Standard deviation

### Interspecific comparison in degree of bioerosion

The degree of bioerosion for *S. rivulatus* was the greatest (1.41–2.66 times greater than the other four species) and the smallest for *S. forsteni* (0.38–0.71 times greater than the other four species) (Table 2). For the other three species (*C. sordidus*, *C. bowersi* and *S. niger*), degree of bioerosion for *C. sordidus* was greater or for *S. niger* (1.25 times greater) whereas the degree of bioerosion was almost equal between *C. bowersi* and *S. niger*. The differences in degree of bioerosion were relatively similar among *C. sordidus*, *C. bowersi* and *S. niger* (0.75 times–1.19 times greater for each species comparison).

**Table 2:** Interspecific comparison of the degree of bioerosion among the five parrotfish species represented by rates of focal species to another species.

Focal species	Compared species	GAI (times)	Feeding rate (times)	Degree in bioerosion (GAI × Feeding rate) (times)
*Chlorurus sordidus*	*C. bowersi*	1.10	1.13	1.25
	*S. rivulatus*	1.76	0.40	0.71
	*S. niger*	2.81	0.47	1.33
	*S. forsteni*	2.81	0.67	1.89
*C. bowersi*	*C. sordidus*	0.91	0.88	0.80
	*S. rivulatus*	1.59	0.36	0.57
	*S. niger*	2.55	0.42	1.07
	*S. forsteni*	2.55	0.59	1.51
*Scarus rivulatus*	*C. sordidus*	0.57	2.48	1.41
	*C. bowersi*	0.63	2.81	1.76
	*S. niger*	1.60	1.18	1.88
	*S. forsteni*	1.60	1.66	2.66
*S. niger*	*C. sordidus*	0.36	2.11	0.75
	*C. bowersi*	0.39	2.38	0.94
	*S. rivulatus*	0.63	0.85	0.53
	*S. forsteni*	1.00	1.41	1.41
*S. forsteni*	*C. sordidus*	0.36	1.49	0.53
	*C. bowersi*	0.39	1.69	0.66
	*S. rivulatus*	0.63	0.60	0.38
	*S. niger*	1.00	0.71	0.71

**Notes.**

GAI Grazing ability index

## Discussion

### Grazing ability index (GAI) to estimate interspecific difference in grazing ability

The present study is the first trial to design a new index of parrotfish grazing ability using stomach contents and to estimate interspecific differences in grazing ability among multiple parrotfish species using the new index. The GAI can be considered as an indicator to enable comparison of the interspecific difference in grazing ability among multiple parrotfish species.

### Interspecific difference in GAI in relation to jaw-lever mechanics

This study is the first study to estimate parrotfish grazing ability in an Okinawan coral reef. Furthermore, although the grazing abilities of *Chlorurus sordidus*, *Scarus rivulatus* and *S. niger* were estimated by previous studies in the Great Barrier Reef and the Red Sea (e.g., Lokrantz et al., 2008; Bonaldo & Bellwood, 2008; Alwany, Thaler & Stachowitsch, 2009), the grazing abilities for *C. bowersi* and *S. forsteni* are first estimated in the present study. The present study also showed the degree of increase in GAI with FL was different among the species. It is suggested that the interspecific differences in GAI among the five species was explained by the interspecific differences in jaw-lever mechanics and relative weight of the adductor mandibulae. Two excavator species (*C. sordidus* and *C. bowersi*) have protrusive-shape teeth and jaw-lever mechanics that produce a greater biting force for both upper and lower jaws than the other three species (*S. rivulatus*, *S. niger* and *S. forsteni*). These two species also showed greater weight of adductor mandibulae, contributing to produce a greater biting force. As a result, the GAI would increase with the increase in FL. In contrast, two scraper species (*S. niger* and *S. forsteni*) have flat-shape teeth and jaw-lever mechanics producing a lower biting force for both upper and lower jaws as well as smaller weight of adductor mandibulae. Consequently, GAI of the two species would be smaller than the other three species (*C. sordidus*, *C. bowersi* and *S. rivulatus*) and GAI would not dramatically increase despite the increase in FL. The present study firstly demonstrated that GAI of *S. rivulatus* was greater than the other two scraper species (*S. niger* and *S. forsteni*), although *S. rivulatus* is categorized into scrapers (Bellwood & Choat, 1990). *S. rivulatus* has jaw-lever mechanics for upper jaws that produces the larger biting force among the three scraper species. Although jaw-lever mechanics for the lower jaw was not significantly different among *C. sordidus*, *C. bowersi* and *S. rivulatus*, the relative weight of adductor mandibulae was significantly less among these three species. Thus, it is suggested that *S. rivulatus* produce an intermediate biting force among the five species. Since the genus *Scarus* consists of 18 species in Okinawa and have various morphological characteristics (Nakabo, 2000), interspecific differences in GAI in further multiple species should be conducted in the future study.

### Interspecific comparison in feeding behavior and degree of bioerosion

Feeding rate and foray size for *S. rivulatus* and *S. niger* was significantly greater than *C. sordidus* and *C. bowersi*. This tendency was consistent with other previous studies indicating that the feeding rates and foray size for scrapers was greater than excavators (reviewed in Bonaldo, Hoey & Bellwood, 2014). Among the scrapers, the feeding rates and foray size for *S. forsteni* was smaller than the other two species. The present study estimated the interspecific difference in degree of bioerosion using GAI and feeding rate. Overall, the degree of bioerosion for *S. rivulatus* was the greatest and the smallest for *S. forsteni*. The degree of bioerosion of *C. sordidus* was greater than for *S. niger* whereas almost the same between *C. bowersi* and *S. niger*. These results suggest that although the GAI was lower for scrapers, scrapers showed greater feeding rates and as a consequence, degree of bioerosion would be greater than excavators in some cases (e.g., *S. rivulatus* in the present study).

The results of the present study suggest the importance of various sizes of parrotfishes with diverse feeding modes. Smaller-sized individuals would contribute to the removal of organic matter (e.g., epilithic algae) on the substrates in a less destructive manner. In contrast, larger-sized individuals, especially for excavators, would contribute to the removal of both organic matter and calcic substrates in a more destructive manner. In some cases such as *S. rivulatus*, which has an intermediate GAI and higher feeding rate, would more effective contribution to bioerosion. Since it has been suggested that increased fishing pressure leads to a decrease in the mean size of parrotfishes (Vallès & Oxenford, 2014; Vallès, Gill & Oxenford, 2015), effective protection of various sizes as well as the various species of parrotfish on reefs should be conducted to maintain a healthy ecosystem balance and resilience in coral reefs.

##  Supplemental Information

10.7717/peerj.2425/supp-1 Figure S1 Jaw-lever mechanics of parrotfishes Observation was based on Bellwood & Choat (1990). An example for Chlorurus sordidus is shown. In (A), yellow arrows show the pulling direction of tendons for jaw closing. In (B), blue and red letters (fulcrum, effort and load) represent the three kinetic points for upper jaw and lower jaw, respectively.

10.7717/peerj.2425/supp-2 Data S1 Figure 4 raw data

10.7717/peerj.2425/supp-3 Data S2 Figure 5 raw data

10.7717/peerj.2425/supp-4 Data S3 Figure 6 raw data

10.7717/peerj.2425/supp-5 Data S4 Figure 7 raw data

10.7717/peerj.2425/supp-6 Data S5 Figure 8 raw data

## Additional Information and Declarations

### Field Study Permissions

The following information was supplied relating to field study approvals (i.e., approving body and any reference numbers): The sampling procedure was approved by Okinawa prefectural government fisheries coordination regulation No. 41, which permits capture of marine fishes on Okinawan coral reefs for scientific purposes.

### Data Availability

The following information was supplied regarding data availability: The raw data has been supplied as Supplemental Files.
