# Supplementary figures and images for: Parrotfish grazing ability: interspecific differences in relation to jaw-lever mechanics and relative weight of adductor mandibulae on an Okinawan coral reef
Source: PeerJ. 2016 Sep 1;4:e2425. doi: 10.7717/peerj.2425 (PMC5012275; doi:10.7717/peerj.2425)

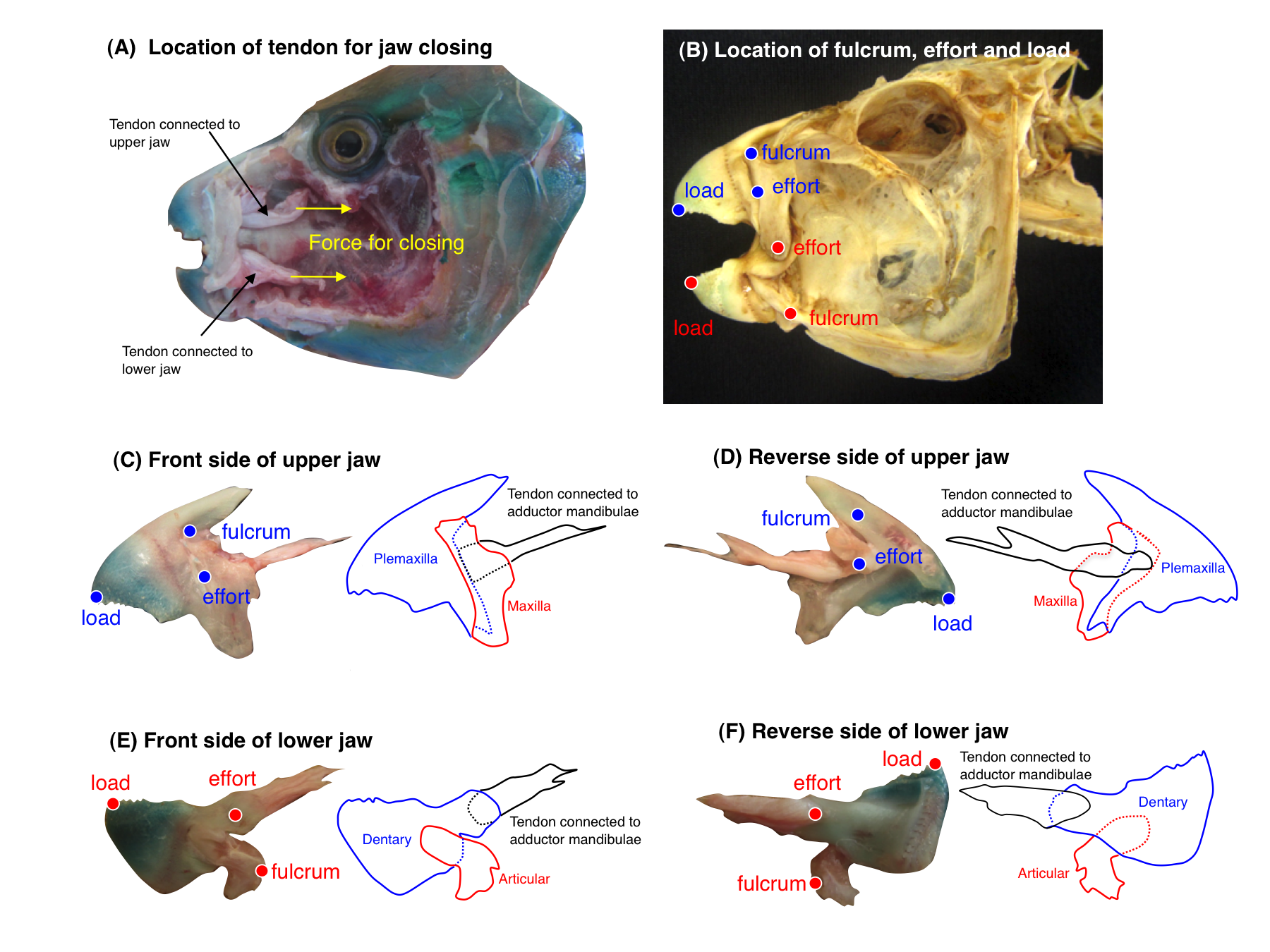

Supplement: Figure S1 — Observation was based on Bellwood & Choat (1990). An example for Chlorurus sordidus is shown. In (A), yellow arrows show the pulling direction of tendons for jaw closing. In (B), blue and red letters (fulcrum, effort and load) represent the three kinetic points for upper jaw and lower jaw, respectively. [file peerj-04-2425-s001.png]
